# Supplementary material for: A zircon case for super-wet arc magmas
Source: Nat Commun. 2024 Oct 17;15:8982. doi: 10.1038/s41467-024-52786-5 (PMC11487280; doi:10.1038/s41467-024-52786-5)
Supplement: Supplementary file 1 — Supplementary Information [file 41467_2024_52786_MOESM1_ESM.pdf]

Supplementary Figures for

A zircon case for super-wet arc magmas

C Nathwani, J Blundy, SJE Large, JJ Wilkinson, Y Buret, MA Loader, L Tavazzani, C Chelle-Michou

*Corresponding author. Email: chetan.nathwani@eaps.ethz.ch

This supplementary file includes:
Figures S1 to S10

Figures

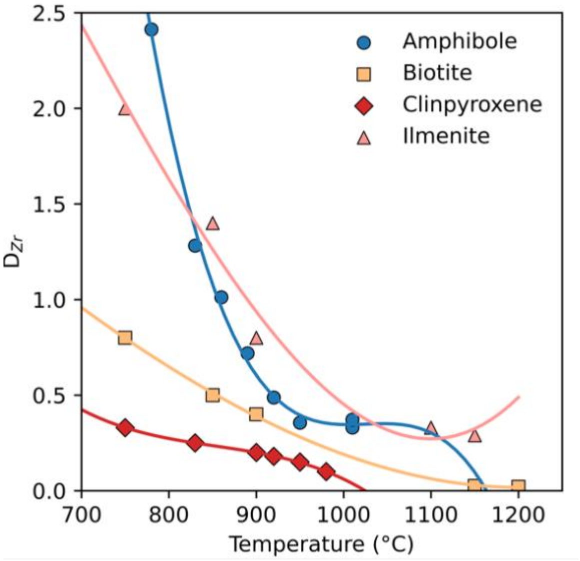

Figure S1. Relationship between temperature and D_{Zr} for different mineral phases (symbols) and the fit of the polynomial regression (curves) for each mineral. The sources of the data are (ref.[1]) for amphibole (ref.[2]) for clinopyroxene and the GERM database for biotite and ilmenite. For plagioclase, D_{Zr} was calculated using the parameterisation of ref.[3].

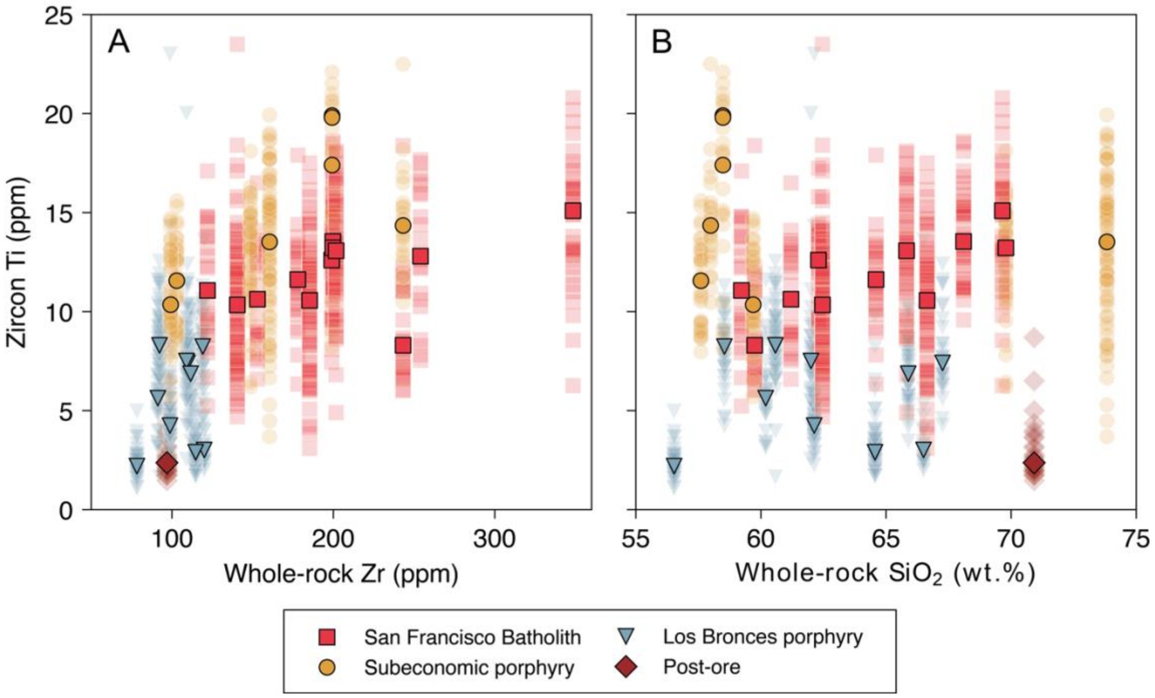

Figure S2. Zircon Ti as a function of host rock (A) Zr and (B) SiO_2. Translucent symbols are zircon Ti concentrations in individual crystals and opaque symbols indicate the mean Ti concentration for each sample. Low zircon Ti contents are found in samples with low bulk-rock Zr. No correlation is present between degree of melt differentiation (SiO_2) and zircon Ti contents.

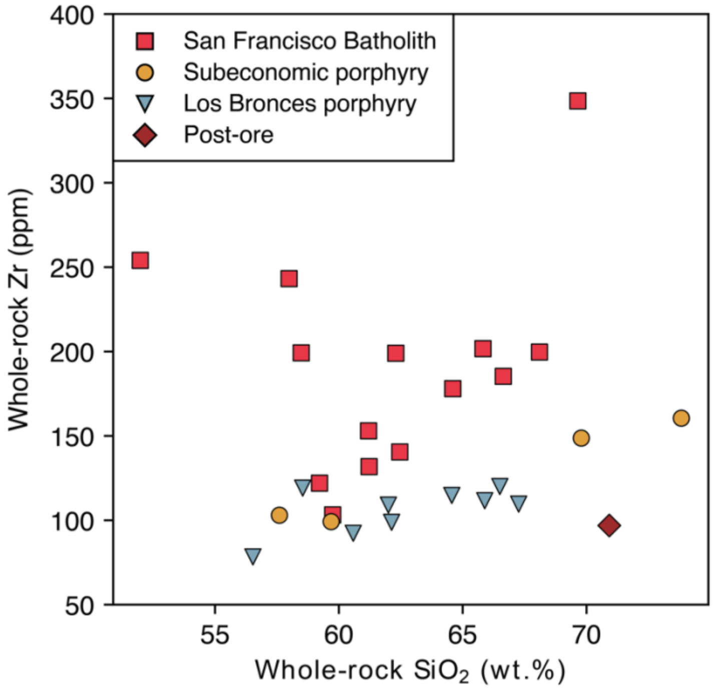

Figure S3. Whole-rock Zr as a function of whole-rock SiO_2 indicating the high and variable concentrations in the San Francisco Batholith and low, invariant concentrations for the Los Bronces porphyry rocks.

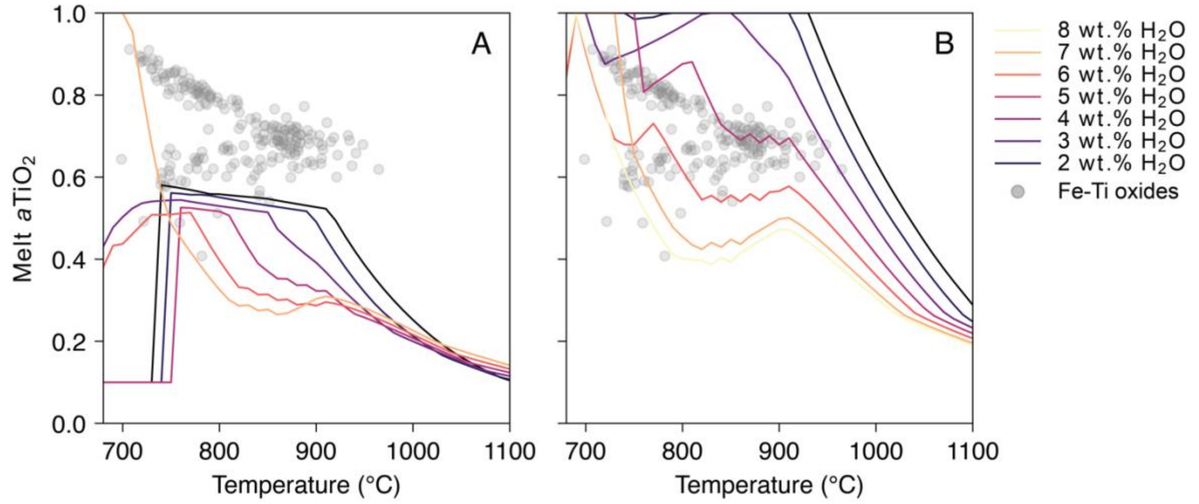

Figure S4. A comparison of melt aTiO$_2$ calculated from (a) rhyolite-MELTS and (b) the rutile solubility model of Borisov and Aranovich[4] for different water contents. Grey symbols show a compilation of aTiO$_2$ for arc-related volcanic systems sourced calculated from Fe-Ti oxide pairs from[5]. Rhyolite-MELTS appears to underestimate aTiO$_2$ whereas the output of Borisov and Aranovich[4] appears to better reproduce natural data. For rhyolite-MELTS with 8 wt.% initial H_2O, the model failed to calculate aTiO$_2$.

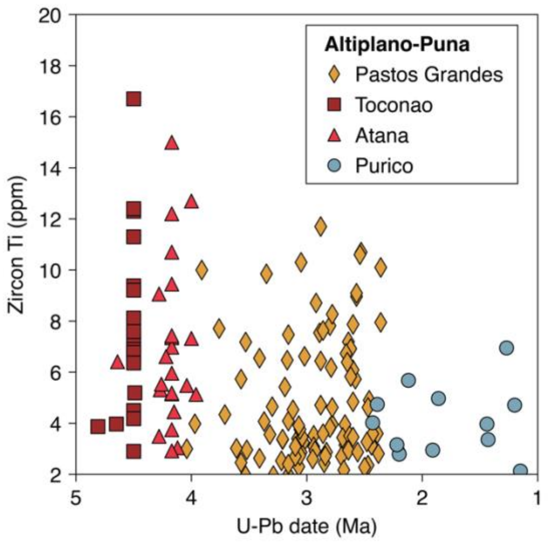

Figure S5. Zircon Ti over time in the Altiplano-Puna Volcanic Complex (Central Andes) over time demonstrating a trend of generally decreasing Ti. Data are from refs.[6,7].

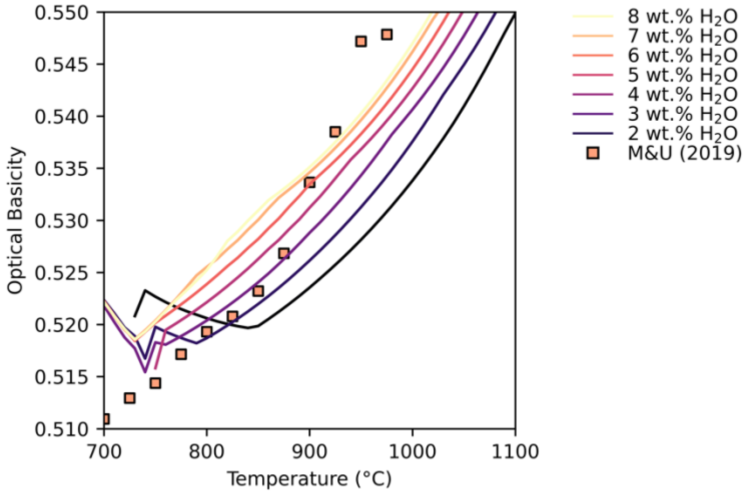

Figure S6. Optical basicity versus temperature for rhyolite-MELTS simulations with different initial H2O concentrations. Symbols show the compositions of glasses in the experimental study of ref.[8] which is based on the same starting composition as the rhyolite-MELTS modelling. The rhyolite-MELTS modelled melts exhibit an inflection at lower temperatures which is not observed in the experimental study. Normal behavior of melt composition is seen at temperatures greater than 850°C and therefore the trend is extrapolated below 850°C to correct for the deviation from typical melt behavior.

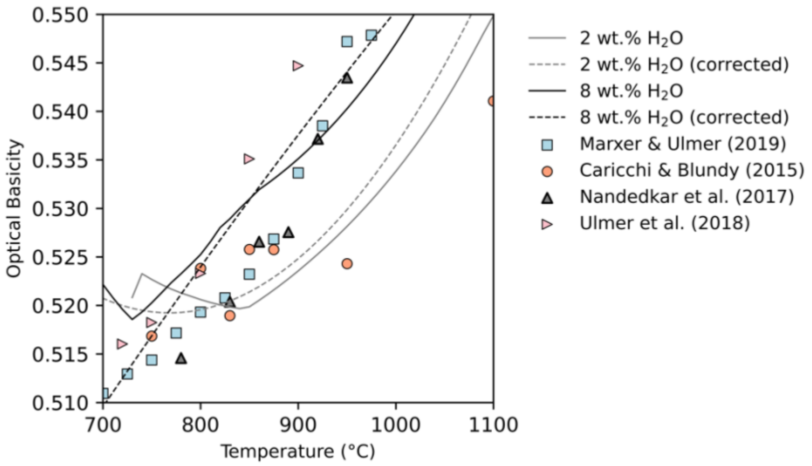

Figure S7. Relationship between temperature and optical basicity from experimental glasses from a range of different experimental studies[8–11]. Curves show the trends of rhyolite-MELTS models for 2 wt.% (gray) and 8 wt.% (black) for the uncorrected (solid) and corrected (dashed) melt compositions.

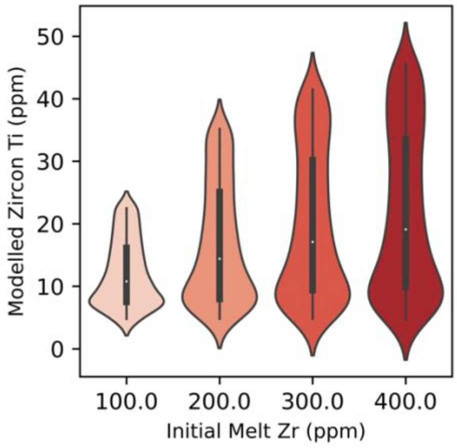

Figure S8. Distributions of modelled Ti-in-zircon as a function of initial melt Zr at 200 MPa with 4 wt.% H_2O.

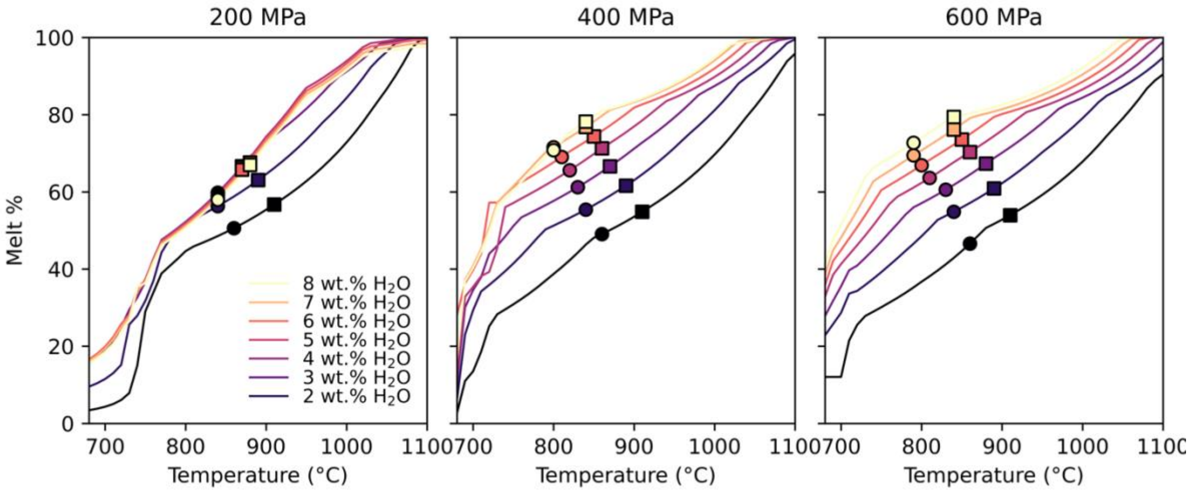

Figure S9. A comparison of rhyolite-MELTS models of cooling of an andesitic magma for different pressures showing melt percentage versus temperature.

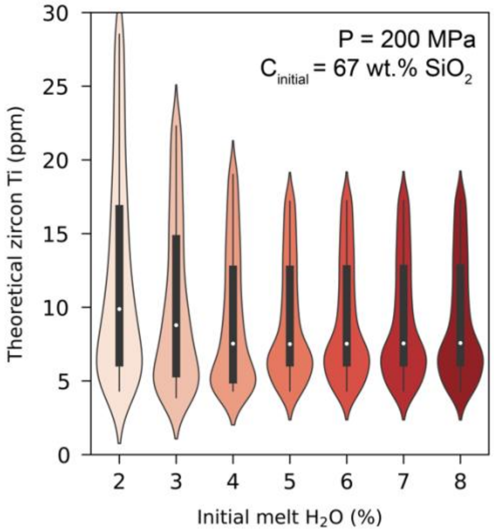

Figure S10. Modelled zircon Ti concentrations as a function of initial water contents using a more evolved starting composition (Pinatubo dacite). Low, homogenous zircon Ti concentrations cannot be produced with a more evolved composition at shallow pressures.

References

1. Nandedkar, R. H., Hürlimann, N., Ulmer, P. & Müntener, O. Amphibole–melt trace element partitioning of fractionating calc-alkaline magmas in the lower crust: an experimental study. *Contributions to Mineralogy and Petrology* **171**, 71 (2016).
2. Bédard, J. H. Parameterizations of calcic clinopyroxene—Melt trace element partition coefficients. *Geochemistry, Geophysics, Geosystems* **15**, 303–336 (2014).
3. Bédard, J. H. Trace element partitioning in plagioclase feldspar. *Geochimica et Cosmochimica Acta* **70**, 3717–3742 (2006).
4. Borisov, A. & Aranovich, L. Rutile solubility and TiO2 activity in silicate melts: An experimental study. *Chemical Geology* **556**, 119817 (2020).
5. Ghiorso, M. S. & Gualda, G. A. R. A method for estimating the activity of titania in magmatic liquids from the compositions of coexisting rhombohedral and cubic iron–titanium oxides. *Contrib Mineral Petrol* **165**, 73–81 (2013).
6. Kern, J. M. *et al.* Geochronological imaging of an episodically constructed subvolcanic batholith: U-Pb in zircon chronochemistry of the Altiplano-Puna Volcanic Complex of the Central Andes. *Geosphere* **12**, 1054–1077 (2016).
7. Kaiser, J. F., de Silva, S., Schmitt, A. K., Economos, R. & Sunagua, M. Million-year melt–presence in monotonous intermediate magma for a volcanic–plutonic assemblage in the Central Andes: Contrasting histories of crystal-rich and crystal-poor super-sized silicic magmas. *Earth and Planetary Science Letters* **457**, 73–86 (2017).
8. Marxer, F. & Ulmer, P. Crystallisation and zircon saturation of calc-alkaline tonalite from the Adamello Batholith at upper crustal conditions: an experimental study. *Contributions to Mineralogy and Petrology* **174**, 84 (2019).
9. Caricchi, L. & Blundy, J. Experimental petrology of monotonous intermediate magmas. *Geological Society, London, Special Publications* **422**, 105–130 (2015).
10. Ulmer, P., Kaegi, R. & Müntener, O. Experimentally Derived Intermediate to Silica-rich Arc Magmas by Fractional and Equilibrium Crystallization at 1·0 GPa: an Evaluation of Phase Relationships, Compositions, Liquid Lines of Descent and Oxygen Fugacity. *Journal of Petrology* **59**, 11–58 (2018).
11. Nandedkar, R. H., Ulmer, P. & Müntener, O. Fractional crystallization of primitive, hydrous arc magmas: an experimental study at 0.7 GPa. *Contributions to Mineralogy and Petrology* **167**, 1–27 (2014).
