# Supplementary material for: A zircon case for super-wet arc magmas
Source: Nat Commun. 2024 Oct 17;15:8982. doi: 10.1038/s41467-024-52786-5 (PMC11487280; doi:10.1038/s41467-024-52786-5)
Supplement: Supplementary file 3 — Description of Additional Supplementary Files [file 41467_2024_52786_MOESM3_ESM.pdf]

<u>Description of Additional Supplementary Files</u>

Supplementary Data S1: LA-ICP-MS analytical metadata
Supplementary Data S2: Los Bronces district zircon LA-ICP-MS data
Supplementary Data S3: Los Bronces whole-rock data
Supplementary Data S4: Reference materials for zircon U-Pb geochronology
Supplementary Data S5: Reference materials for zircon trace element analyses
